# The Public Health Impact and Policy Implications of Online Support Group Use for Mental Health in Singapore: Cross-Sectional Survey

**DOI:** 10.2196/18114

**Published:** 2020-08-04

**Authors:** Kumarasan Roystonn, Janhavi Ajit Vaingankar, Boon Yiang Chua, Rajeswari Sambasivam, Saleha Shafie, Anitha Jeyagurunathan, Swapna Verma, Edimansyah Abdin, Siow Ann Chong, Mythily Subramaniam

**Affiliations:** 1 Research Division Institute of Mental Health Singapore Singapore; 2 Clinical Education Office of Education Duke-NUS Medical School Singapore Singapore; 3 Lee Kong Chian School of Medicine Nanyang Technology University Singapore Singapore; 4 Saw Swee Hock School of Public Health National University of Singapore Singapore Singapore

**Keywords:** online support group, internet, self-help, mental health treatment, mental illness, public health

## Abstract

**Background:**

The wide mental health treatment gap continues to pose a global and local public health challenge. Online support groups are on the rise and could be used to complement formal treatment services for mental health.

**Objective:**

This study aimed to examine the prevalence of online support group use and explore factors associated with the use in the general population using data from a national cross-sectional mental health survey in Singapore.

**Methods:**

Singapore residents aged 18 years and above participated in a nationally representative household survey in which the World Health Organization Composite International Diagnostic Interview 3.0 was administered by trained interviewers to examine the use of online support groups for mental health. Multiple logistic regressions were used to analyze the association of online support group use with various sociodemographic and health factors.

**Results:**

A total of 6110 respondents with complete data were included in this study. Overall, 10 individuals per 1000 adults (1%) reported seeking help from online support groups for their mental health problems. Compared to younger adults (those aged 18 to 34 years) and those with university education, individuals aged 50 to 64 years (*P*<.001; OR 0.1, 95% CI 0.0-0.3) and those with preuniversity qualifications (*P*=.02; OR 0.1, 95% CI 0.0-0.8) were less likely to use online support groups for mental health, respectively. Participants with a Diagnostic and Statistical Manual of Mental Disorders, Fourth Edition (DSM-IV) mental disorder were 6.8 times more likely (*P*<.001; 95% CI 3.0-15.4) to use an online support group; in particular, individuals with major depressive disorder (*P*<.001; OR 5.4, 95% CI 2.1-13.8) and obsessive compulsive disorder (*P*=.01; OR 3.5, 95% CI 1.3-9.7) were more likely to use an online support group for their mental health.

**Conclusions:**

Online support groups could be used to complement formal treatment services, especially for mood and anxiety-related disorders. As online support group use for mental health issues may be more prevalent among younger people, early detection and accurate information in online support groups may guide individuals toward seeking professional help for their mental health problems.

## Introduction

The internet is increasingly recognized as a valuable self-help resource for individuals. Online support groups in the form of web-based forums, discussion boards, and chat rooms are fast emerging as a popular and accessible means to provide individuals the opportunity to reach out to peers without geographical restrictions, share personal health experiences, and receive emotional support for mental health issues [1]. Disease-specific online mental health groups are especially important because research has shown that people prefer them to other types of support networks or internet features as they provide peer support (support from people going through the same experience) and there is a greater degree of empathy in such mental health forums and chat rooms [2,3]. The increasing use of online and mobile technologies for mental health peer support has been termed “digital peer support”; this is defined by researchers as “live or automated peer support services delivered through technology media such as peer-to-peer networks on social media, peer-delivered interventions supported by smartphone apps, and asynchronous and synchronous technologies” [1]. They also provide other essential elements important for coping, including the following: empowerment, information, advice, a sense of control, and a sense of being able to help others [4,5]. Thus, mental health peer support in online support groups facilitates the sharing of personal experiences, enables information exchange on the day-to-day management of disease, and is a source of advice on resolving some of the most psychologically difficult issues among peers with similar mental health problems and comorbid conditions [6]. Given approximately 450 million people with mental illness worldwide, mental health is deemed a major public concern today [7]. Over one-third of people in most countries experience at least one mental disorder in their lifetime. For example, prevalence estimates suggest 40.9%, 37.5%, and 48.6% of people in the Netherlands, Canada, and United States meet the criteria for a mental disorder at some time in their life, respectively [8]. In Singapore, a city-state in Southeast Asia, 1 in 7 adults are reported to have at least one mood, anxiety, or alcohol use disorder during their lifetime [9]. Researchers estimate that mental health–related online support groups are accessed almost daily, especially in countries with high internet availability [10]. The primary function of such online support groups is to provide a space where mutual support is given and received by anyone affected by mental health issues. People who feel stigmatized about their mental health issues and reluctant to seek professional help in person are more likely to use these online communities privately and anonymously [11]. Many systematic reviews and randomized controlled trials have reported that online support groups have great potential for helping people manage a variety of mental health problems [12,13]. The evidence suggests that the use of these online support groups reduces social isolation, psychological distress, and empowers individuals with knowledge about mental health, treatment options, and symptom management [14-16]. However, national prevalence estimates for online support group use among the general population is limited [17]. Population-based studies and systematic reviews focused on the use of the internet for mental health information, but did not consider the use of online support groups [18-20]. A highly publicized study from the Pew Research Center reported that 510 out of 3001 (17%) internet users in the United States had contacted health-related online support groups [21]. However, this survey accounted for chronic physical conditions, and did not include the use of online support groups for mental health problems. In contrast, a meta-analysis of US population data [22] indicated that 2.7 to 3.3 people per 1000 community-dwelling adults were likely to have visited online mental health support groups or chat rooms, and the figure remained stable despite annual reports of an upward trend in online activities and internet access across the United States [23]. Additionally, while research has shown that the proportion of people in the general population accessing online support groups for mental health may be small, the proportion of people with diagnosed mental disorders accessing mental health support groups and chat rooms online remains relatively high. Researchers report that online support groups are particularly popular among individuals living with a mental disorder worldwide [12,22-25]. Depression support groups and chat rooms have been reported to be one of the most common online support groups sought on the internet [26,27]. In Germany, Diskussionsforum Depression [28] is an established online community for those dealing with specific psychological problems. Other well-known online support groups, such as Care Opinion [29] in the United Kingdom, developed an online community for individuals to share their personal stories including experiences with depression and other mental health problems. People living with bipolar disorder have also reportedly found online chat rooms and web-based social support groups helpful, citing camaraderie, cohesion with their online peers, and feeling empowered to cope with their condition [30]. However, research on the prevalence and use of mental health social support communities in non-Western populations is scant.

Singapore has a resident population of just over 5.6 million people. The literacy rate of Singapore’s population is 97.3%. Overall, 84% of the population (4.83 million people) are internet users, and over 4.4 million are active on social media, discussion forums, and various other internet communication tools [31]. Research has consistently shown that Asians prefer to seek help from less formal sources for their mental health problems, which could potentially include support groups and chat rooms online [32-34]. Help-seeking behaviors for mental health problems may also be largely influenced by cultural influences in Asian societies. Stigma may be a deterrent and has been identified as a barrier to service use in Asian societies [35,36]. The use of online support groups for mental health support might be of particular local interest due to its capacity for providing easy access, anonymity, and the potential to overcome perceived stigma in seeking help for mental health issues. In this way, online mental health support communities offer a valuable source of peer advice and may provide the stimulus to seek professional help. Online support groups may have the public health potential to help individuals manage their mental health problems and improve treatment-seeking [37]. Against this background, this study seeks to broadly explore the use of online support groups in Singapore and the factors associated with their use by using population-based and nationally representative data.

## Methods

### Survey Population and Subjects

Data for this study were obtained from the Singapore Mental Health Study (SMHS) 2016, a population-based cross-sectional epidemiological survey of noninstitutionalized respondents aged 18 years and above, primarily conducted to establish lifetime and 12-month prevalence of mental disorders, as well as the current use of mental health services. Of the 6126 survey respondents, this study uses data drawn from a subsample of 6110 subjects who responded to a question regarding the use of online support groups for mental health. The study was conducted from 2016 to 2018, and achieved a response rate of 69.5% among eligible adults. The sampling frame was based on a national administrative database of all citizens and permanent residents of Singapore. A probability random sample was selected using a disproportionate stratified sampling design of 16 strata defined with respect to ethnicity (Chinese, Malay, Indian, Others) and age groups (18 to 34, 35 to 49, 50 to 64, and ≥65 years). Older adults (≥65 years) and ethnic minority groups (Malays and Indians) were oversampled to ensure sufficient sample size to improve the reliability of estimates for subgroup analysis. 

### Procedure

A printed invitation letter was sent to each subject, followed by a household visit to obtain their agreement to participate in the survey. Trained interviewers from a survey research company conducted face-to-face interviews with the respondents who agreed to participate in the study. Respondents were asked to choose their preferred language (questionnaires were available in English, Chinese, and Malay) before the interviewer initiated any study-related procedures. Residents who were living outside of Singapore, institutionalized, or hospitalized at the time of the survey, those who were not contactable due to an incomplete or incorrect address, and those who were unable to complete the interview in one of the specified languages (English, Chinese, or Malay) were excluded from the survey. The SMHS 2016 study methodology has been reported in much greater detail elsewhere [38].

The study was approved by the relevant institutional ethics committee, the National Healthcare Group Domain Specific Review Board. All participants provided written informed consent. For those aged <21 years, written informed consent was also obtained from their parent or legally acceptable representative.

### Measures

#### World Health Organization Composite International Diagnostic Interview

A standardized computer-assisted version of the Composite International Diagnostic Interview version 3.0 (CIDI 3.0) [39] was the primary diagnostic instrument used in this study. The CIDI 3.0 has established psychometric properties and has been used widely in psychiatric epidemiological studies. The Services module within the CIDI 3.0 instrument broadly assesses help-seeking sources used for mental health, which included the following question to establish the prevalence of online support group use: “Did you ever use an internet support group or chat room to get help for problems with your emotions or nerves?”

For each mental disorder, a screening section was administered to all respondents. All participants answering positively to a specific screening question were then referred to the respective diagnostic section of the questionnaire. Given practical survey limitations of time and burden on the participant, only selected modules from the CIDI were included based on input from a stakeholder board. This board included representatives from various stakeholders (Ministry of Health, voluntary organizations working with mentally ill clients, clinicians, sociologists, and representatives from the major ethnic groups in Singapore) who advised the study team on the modules considered locally relevant [38]. The disorders were selected based on the main consideration of what would be of relevance to the country and its policy makers and service providers in the wake of the National Mental Health Blueprint and Policy in Singapore. This study was guided by a review of published literature and national reports. Through these processes, the following mental disorders were assessed based on the diagnostic criteria of the Diagnostic and Statistical Manual of Mental Disorders, Fourth Edition (DSM-IV) [40] and analyzed for this study: major depressive disorder (MDD), dysthymia, and bipolar disorder (mood disorders); generalized anxiety disorder (GAD) and obsessive compulsive disorder (OCD; anxiety disorders); alcohol abuse and alcohol dependence (alcohol use disorders). The CIDI 3.0 interviews lasted approximately 1.5 to 2 hours.

#### Chronic Physical Conditions

A range of chronic physical conditions were reported by participants on the modified version of the CIDI chronic conditions checklist. The physical conditions included on the checklist were based on their prevalence in Singapore as per local statistics. Participants were asked if they had been clinically diagnosed with the following list of health conditions: hypertension, hyperlipidemia, diabetes, asthma, arthritis or rheumatism, back problems (of disc or spine), migraine headaches, stroke or major paralysis; heart disease including heart attack, coronary heart disease, angina, or congestive heart failure; chronic inflamed bowel problems (eg, stomach ulcer, enteritis, or colitis), neurological disorders (epilepsy, convulsions, and Parkinson disease), thyroid diseases, kidney failure, chronic lung diseases (chronic bronchitis or emphysema), and cancer.

#### Sociodemographic Information

Sociodemographic data collected from participants included age, gender, ethnicity, income, education, marital status, and employment status.

### Statistical Analysis

To ensure that findings would be nationally representative, sample estimates were weighted to account for oversampling and nonresponse, and were poststratified for age group and ethnic distributions between the study sample and the Singapore resident population in 2014. Descriptive statistics were performed to describe the sociodemographic profile of the study population. Binary logistic regressions were performed where the outcome variable (use of online support groups) was treated as binary (Yes=1, No=0) and the mental health predictors were similarly treated as binary variables (Yes=1, No=0), where the values (1 or 0) indicated the presence or absence of the condition based on the DSM-IV criteria for the disorder. Multiple logistic regressions were then used to investigate the associations between the outcome (use of online support groups) and each sociodemographic and health condition as an independent variable, controlling for the presence of all other sociodemographic differences in gender, age, ethnicity, marital status, education, employment, and income. The estimates obtained from the analyses were adjusted odds ratios (OR) and statistical significance was established at *P*<.05. All statistical analyses were carried out using SAS software (version 9.3; SAS Institute) [41].

## Results

### Study Sample Characteristics

The sociodemographic and clinical characteristics of the sample (n=6110) are presented in Multimedia Appendix 1. Weighted analysis of the population sample showed an estimated 10 individuals per 1000 adults (1%, 53/6110) had sought help from an online support group for their mental health problems.

Of the 53 respondents who used online support groups, weighted estimates show that 56.9% (n=29) were female, 76.1% (n=17) were of Chinese ethnicity, 79.4% (n=40) were never married, 70.6% (n=36) were employed, and 82.2% (n=45) were aged 18 to 34 years. In total, 58.2% (n=25) of the online support group users were represented by those with mental illness (Multimedia Appendix 2).

### Sociodemographic and Clinical Correlates of Online Support Group Participation

Table 1 presents the results of the logistic regression analysis performed to examine the sociodemographic and clinical correlates of participation in online support groups. Compared to participants aged 18 to 34 years, individuals aged 50 to 64 years (*P*<.001; OR 0.1, 95% CI 0.0-0.3) were less likely to use online support groups for mental health. Compared to participants with a university education, those with preuniversity or junior college education (*P*=.02; OR 0.1, 95% CI 0.0-0.8) were also less likely to use an online support group for mental health. Compared to those without a mental disorder, the odds of use of an online support group was 6.8 times (*P*<.001; 95% CI 3.0-15.4) higher for participants with a mental disorder in this sample.

In Table 2, the associations between types of mental disorders and online support group participation in the sample indicate that individuals with MDD (*P*<.001; OR 5.4, 95% CI 2.1-13.8) or OCD (*P*=.01; OR 3.5, 95% CI 1.3-9.7) are more likely to use online support groups for mental health support.

**Table 1:** Sociodemographic and clinical correlates of online support group users.

Parameters		OR^a^	95% CI	*P* value
**Age group (years)**				
	18-34	Reference	N/A^b^	N/A
	35-49	0.5	0.1-1.7	.25
	50-64	0.1	0.0-0.3	<.001
	≥65^c^	—^d^	—	—
**Gender**				
	Female	Reference	N/A	N/A
	Male	0.5	0.2-1.2	.11
**Ethnicity**				
	Chinese	Reference	N/A	N/A
	Malay	0.9	0.4-2.1	.80
	Indian	0.8	0.4-1.8	.56
	Other	1.1	0.4-3.5	.83
**Marital status**				
	Married	Reference	N/A	N/A
	Never married	2.6	0.9-7.7	.09
	Divorced or separated	0.6	0.1-3.8	.62
	Widowed^c^	—	—	—
**Education**				
	University	Reference	N/A	N/A
	Primary and below^c^	—	—	—
	Secondary	0.4	0.1-1.9	.26
	Preuniversity/junior college	0.1	0.0-0.8	.02
	Vocational institute/Institute of Technical Education	0.7	0.2-2.5	.56
	Diploma	0.7	0.2-2.2	.57
**Employment**				
	Employed	Reference	N/A	N/A
	Economically inactive^e^	1.2	0.4-3.2	.74
	Unemployed	2.0	0.6-6.9	.28
**Monthly household income (SGD $)^f^**				
	<2000	Reference	N/A	N/A
	2000-3999	1.5	0.4-6.0	.60
	4000-5999	1.3	0.3-5.0	.73
	6000-9999	0.6	0.1-2.5	.44
	≥10,000	0.9	0.2-5.0	.91
**Any mental disorder^g^**				
	No	Reference	N/A	N/A
	Yes	6.8	3.0-15.4	<.001

^a^The odds ratio was derived using multiple logistic regressions after controlling for sociodemographic variables.

^b^N/A: not applicable.

^c^Due to a lower number of cases, the regression coefficient was not estimated.

^d^Not available.

^e^This group includes homemakers, students, and retirees/pensioners.

^f^SGD: Singapore Dollar.

^g^The participant has at least one of the mental disorders assessed by the Composite International Diagnostic Interview.

**Table 2:** Association of mental disorders and online support group use in the population.

Mental disorder	OR^a^	95% CI	*P* value
Major depressive disorder	5.4	2.1-13.8	<.001
Dysthymia	0.4	0.0-4.8	.46
Bipolar disorder	2.7	0.5-13.9	.23
Generalized anxiety disorder	3.6	0.9-14.1	.06
Obsessive compulsive disorder	3.5	1.3-9.7	.01
Alcohol abuse	2.7	0.1-12.5	.13
Alcohol dependence	0.9	0.0-0.1	.92

^a^The odds ratio was derived using multiple logistic regressions after controlling for sociodemographic variables.

### Sociodemographic and Clinical Correlates of the Psychiatric Population

Multimedia Appendix 3 presents the percentage of online support group users among those with a DSM-IV mental disorder in the population. Among 839 respondents with a mental disorder, 25 (4.3%) of them used an online support group for mental health support.

Table 3 shows that men (*P*=.02; OR 0.3, 95% CI 0.1-0.8), those of Malay ethnicity (*P*=.03; OR 0.2, 95% CI 0.0-0.8), and those aged 50 to 64 years (*P*=.01; OR 0.02, 95% CI 0.0-0.4), were significantly less likely to use online support groups while those who were unemployed (*P*=.02; OR 6.2, 95% CI 1.3-28.2) and living with a comorbid chronic condition (*P*=.04; OR 3.4, 95% CI 1.1-10.7), were more likely to use an online support group for mental health support.

Additionally, Table 4 reveals that after controlling for significant sociodemographic correlates, persons with MDD and OCD were more likely to use an online support group among participants diagnosed with a lifetime mental disorder. The odds of online support group use for participants with MDD and OCD were 4.3 times (*P*<.001; 95% CI 2.1-13.8), and 4.1 times (*P*=.002; 95% CI 1.3-9.7) higher compared to those living with other mental disorders, respectively.

**Table 3:** Sociodemographic and clinical correlates of online support group use among those with any mental disorder.

Demographics		OR^a^	95% CI	*P* value
**Age group (years)**				
	18-34	Reference	N/A^b^	N/A
	35-49	0.5	0.1-3.6	.53
	50-64	0.02	0.0-0.4	.01
	≥65^c^	—^d^	—	—
**Gender**				
	Female	Reference	N/A	N/A
	Male	0.3	0.1-0.8	.02
**Ethnicity**				
	Chinese	Reference	N/A	N/A
	Malay	0.2	0.0-0.8	.03
	Indian	0.4	0.1-1.4	.17
	Other	0.8	0.2-4.3	.81
**Marital status**				
	Married	Reference	N/A	N/A
	Never married	4.4	0.7-28.0	.11
	Divorced or separated	1.1	0.1-11.6	.95
	Widowed^c^	—	—	—
**Education**				
	University	Reference	N/A	N/A
	Primary and below^c^	—	—	—
	Secondary	0.7	0.1-5.5	.74
	Preuniversity/junior college	—	—	—
	Vocational institute/Institute of Technical Education	0.9	0.1-5.7	.88
	Diploma	0.5	0.1-3.2	.48
**Employment**				
	Employed	Reference	N/A	N/A
	Economically inactive^e^	1.4	0.4-5.1	.62
	Unemployed	6.2	1.3-28.2	.02
**Monthly household income (SGD $)^f^**				
	<2000	Reference	N/A	N/A
	2000-3999	0.7	0.1-4.1	.70
	4000-5999	1.2	0.2-6.3	.85
	6000-9999	0.5	0.1-3.5	.47
	≥10,000	0.8	0.1-8.3	.82
**Any chronic condition^g^**				
	No	Reference	N/A	N/A
	Yes	3.4	1.1-10.7	.04

^a^The odds ratio was derived using multiple logistic regressions after controlling for significant sociodemographic correlates.

^b^N/A: not applicable.

^c^Due to a lower number of cases, the regression coefficient was not estimated.

^d^Not available.

^e^This group includes homemakers, students, and retirees/pensioners.

^f^SGD: Singapore dollar.

^g^The participant has at least one of the chronic conditions on the checklist assessed by the Composite International Diagnostic Interview.

**Table 4:** Association of online support group use and type of mental disorder in the psychiatric population.

Mental disorder	OR^a^	95% CI	*P* value
Major depressive disorder	4.3	2.1-13.8	<.001
Dysthymia	0.3	0.0-4.8	.32
Bipolar disorder	4.0	0.5-13.9	.06
Generalized anxiety disorder	3.0	0.9-14.1	.16
Obsessive compulsive disorder	4.1	1.3-9.7	.002
Alcohol abuse	1.7	0.1-12.5	.33
Alcohol dependence	0.6	0.0-0.1	.68

^a^The odds ratio was derived using multiple logistic regressions after controlling for significant sociodemographic correlates.

## Discussion

### Principal Findings

This study analyzed data from a national cross-sectional mental health survey to examine the prevalence of online support group participation (ie, internet support groups or chat rooms) for mental health, and the sociodemographic and health status factors associated with those who were more likely to engage in these activities. Of the 6110 survey participants, an estimated 10 individuals per 1000 adults (1%) had sought help from an online support group for their mental health problems, and individuals who were aged 18 to 34 years or who had a university education were more likely to use online support groups for mental health. Additionally, this study found that a substantial proportion (58.2%) of those using an online support group had met the criteria for a DSM-IV mental disorder. Among those with mental disorders, individuals with MDD and OCD had the highest rates of online support group participation.

### Comparison With Previous Studies

Comparable to other developed countries, more than 80% of the population in Singapore is internet savvy [31]. However, only a small proportion of the sample used an online support group such as an internet support group or chat room to seek help for their mental health issues. In this context, we refer to seeking out peer support in online self-help forums and chat rooms to deal with their mental health problems. Help-seeking in online support groups for mental health support appears to be an infrequent activity in this sample, even though there are many web-based mental health peer support communities and chat rooms in Singapore (eg, web-CHAT [42]). Our estimates are consistent with those reported among general populations elsewhere in the developed world [22,43]. For instance, a meta-analytic survey of household residents in the United States acknowledged that only 0.3% (3 individuals per 1000 adults) had visited support groups or chat rooms for mental health support online [22].

In earlier research, younger individuals and those with tertiary educational qualifications [44,45] were found to seek help for mental health problems on the internet via chats and social groups. Our results mirror previous reports in that those who were younger (versus those aged 50 to 64 years) and university-educated (versus those with preuniversity qualifications) emerge as sociodemographic groups who are more likely to seek help from online support groups for mental health support. Therefore, targeted interventions in these groups, with an emphasis on raising awareness about accessible youth-friendly professional care services offline (as well as online), may lead to more effective prevention and treatment efforts [46].

Additionally, this study found that a substantial proportion (58.2%) of those using an online support group had a diagnosable DSM-IV mental disorder. Griffiths and colleagues [47] previously identified depression support groups on the internet as the most commonly used online support group; this study similarly found that individuals with depression had the highest rates of online support group participation. Online support group participation was also high for individuals with OCD, which holds important implications for psychiatric care providers. Online support groups in the form of internet support groups and chat rooms may offer a valuable source of information and social support for those with mental health problems, particularly mood and anxiety problems. Recent evidence suggests that the wide treatment-seeking gap for mental health continues to pose a local public health challenge [48]. Hence, online support groups could guide individuals to seek professional help for their mental health problems; in addition, they could complement the formal treatment services that patients may receive. Furthermore, health care professionals can explore the impact of online support groups on the individual and provide appropriate support and guidance where needed. Mental health support initiatives can take these findings into consideration when programs are developed and implemented so as to effectively deliver the intended message and services to the targeted audience.

### Strengths and Limitations

A key strength of this study is that it fills an important gap in the extant literature and is among the first to report extensively on nationally representative population data in Singapore. To the best of our knowledge, this study is the first to examine the prevalence and correlates of online support group use for mental health support among the general public in a non-Western society. However, the findings of this study must be considered in view of its limitations. Despite the use of a relatively large population-based survey for analysis, the sample size of those who had used an online support group was rather small. The low rate of online support group participation reported may have been a result of the survey methodology. In this study, participants were asked about their use of internet support groups or chat rooms; however, people may have accessed web forums, mailing lists, or e-blogs to provide or receive online social support or to share mental health experiences. Hence, individuals may not have identified this variety of web communication applications as characteristic of an “internet support group or chat room.” Therefore, the true prevalence of online support group use may have been underestimated. In addition, there are new and emerging online peer groups on social networking sites (eg, Twitter, Facebook), which allow users to exchange information or seek emotional support from peers with similar health issues [49]. The impact of such social networking sites may have been missed in this study. Additionally, it is not known if the use of online support groups results in improved clinical outcomes, which is beyond the scope of this study. Nonetheless, this study established an important starting point. Further research is necessary to investigate the reach of online mental health support communities, potential barriers to local participation in such communities, and the impact of this participation on clinical outcomes. Furthermore, specific questions regarding the types of online social support tools (eg, blogs, message boards, mailing lists, social media sites) sought out by internet users today must be incorporated into survey methodology to gain a deeper understanding of user behavior.

Technology is constantly advancing and the use of technology is changing quickly. Health care delivery systems are beginning to integrate the use of electronic Health (eHealth) and internet tools. In the United Kingdom, United States, Europe, Australia, and New Zealand, mental health services such as online support groups are currently being integrated into the health care delivery system [50]. Online support groups are readily accessible online and internet applications are relatively low-cost. It is increasingly likely that individuals with mental health problems may turn to technology for help [51]. Internet-based support communities have great potential to manage mental health at significantly reduced costs to the health care system. Hence, it is vital that we continue to explore whether and how online support groups can be effectively used in the local health care delivery system for mental health services.

## Appendix

Multimedia Appendix 1 Sample characteristics of study participants and the use of online support groups across sociodemographic groups.

Multimedia Appendix 2 Clinical characteristics of study participants and the use of online support groups.

Multimedia Appendix 3 Online support group utilization among those with a DSM-IV mental disorder.

## Abbreviations

CIDI 3.0: Composite International Diagnostic Interview version 3.0
DSM-IV: Diagnostic and Statistical Manual of Mental Disorders, Fourth Edition
GAD: generalized anxiety disorder
MDD: major depressive disorder
OCD: obsessive compulsive disorder
SMHS: Singapore Mental Health Study
