# Supplementary material for: The Public Health Impact and Policy Implications of Online Support Group Use for Mental Health in Singapore: Cross-Sectional Survey
Source: JMIR Ment Health. 2020 Aug 4;7(8):e18114. doi: 10.2196/18114 (PMC7435627; doi:10.2196/18114)
Supplement: Multimedia Appendix 1 [file mental_v7i8e18114_app1.docx]

|  |  | Online Support Group Users |
| --- | --- | --- |
|  | N (%) | N (%) |
| **Age Group (years)** |  |  |
| 18 – 34 | 1701 (30.5) | 45 (82.2) |
| 35- 49 | 1493 (29.6) | 6 (16.9) |
| 50 – 64 | 1623 (26.9) | 2 (0.8) |
| ≥65 | 1293 (13.1) | —^a^ |
| **Gender** |  |  |
| Female | 3048 (50.4) | 29 (56.9) |
| Male | 3062 (49.6) | 24 (43.1) |
| **Ethnicity** |  |  |
| Chinese | 1777 (75.7) | 17 (76.1) |
| Malay | 1985 (12.5) | 15 (12.1) |
| Indian | 1840 (8.7) | 16 (8.8) |
| Other | 508 (3.1) | 5 (3.0) |
| **Marital Status** |  |  |
| Never married | 1538 (31.0) | 40 (79.4) |
| Married | 3835 (59.8) | 11 (19.5) |
| Divorced or separated | 341 (5.2) | 2 (1.1) |
| Widowed | 396 (4.1) | — |
| **Education** |  |  |
| Primary and below | 1183 (16.2) | — |
| Secondary | 1640 (23.0) | 9 (13.3) |
| Vocational institute/Institute of Technical Education | 507 (6.3) | 8 (9.5) |
| Preuniversity/junior college | 302 (6.0) | 2 (1.4) |
| Diploma | 1023 (19.0) | 19 (31.5) |
| University | 1455 (29.5) | 15 (44.3) |
| **Employment** |  |  |
| Employed | 4048 (72.1) | 36 (70.6) |
| Economically inactive^b^ | 1711 (22.8) | 13 (15.5) |
| Unemployed | 350 (5.2) | 4 (13.9) |
| **Monthly household income (SGD $)^c^** |  |  |
| <2000 | 1139 (16.3) | 5 (7.8) |
| 2000 – 3999 | 1331 (20.1) | 14 (27.7) |
| 4000 – 5999 | 1110 (21.4) | 11 (26.3) |
| 6000 – 9999 | 1000 (21.8) | 11 (16.5) |
| ≥10,000 | 861 (20.4) | 7 (21.8) |

^a^Not available.

^b^This group includes homemakers, students, and retirees/pensioners.

^c^SGD –Singapore Dollar.
