# Supplementary material for: The Public Health Impact and Policy Implications of Online Support Group Use for Mental Health in Singapore: Cross-Sectional Survey
Source: JMIR Ment Health. 2020 Aug 4;7(8):e18114. doi: 10.2196/18114 (PMC7435627; doi:10.2196/18114)
Supplement: Multimedia Appendix 2 [file mental_v7i8e18114_app2.docx]

|  |  | Online Support Group Users |
| --- | --- | --- |
|  | N (%) | N (%) |
| **Any mental disorder^a^** |  |  |
| No | 5271 (86.2) | 28 (41.8) |
| Yes | 839 (13.8) | 25 (58.2) |
| **Mental disorder type** |  |  |
| Major depressive disorder | 343 (6.2) | 13 (31.3) |
| Dysthymia | 26 (0.3) | 1 (0.5) |
| Bipolar disorder | 103 (1.6) | 5 (14.2) |
| Generalized anxiety disorder | 100 (1.6) | 4 (11.4) |
| Obsessive compulsive disorder | 215 (3.5) | 11 (30.7) |
| Alcohol abuse | 245 (4.1) | 3 (13.1) |
| Alcohol dependence | 41 (0.5) | 2 (1.4) |

^a^The participant has at least one of the mental disorders assessed by the Composite International Diagnostic Interview.
