# Supplementary material for: The Public Health Impact and Policy Implications of Online Support Group Use for Mental Health in Singapore: Cross-Sectional Survey
Source: JMIR Ment Health. 2020 Aug 4;7(8):e18114. doi: 10.2196/18114 (PMC7435627; doi:10.2196/18114)
Supplement: Multimedia Appendix 3 [file mental_v7i8e18114_app3.docx]

|  | N | Weighted % |  |
| --- | --- | --- | --- |
| **Online support group use** |  |  |  |
|  |  |  |  |
| Yes^a^ | 25 | 4.3 |  |
| No | 814 | 95.7 |  |

^a^Among those with at least one of the mental disorders assessed by the Composite International Diagnostic Interview.
